# Comparing Pain and Fear Experiences in Pediatric Patients Undergoing Venipuncture and Cannulation Procedures: A Cross‐Sectional Study

**DOI:** 10.1002/hsr2.73063

**Published:** 2026-10-08

**Authors:** Sherzad Khudeida Suleman, Nizar BakirYahya, Stefan Nilsson, Karin Enskär

**Affiliations:** ^1^ Department of Women's and Children's Health Uppsala University Uppsala Sweden; ^2^ College of Nursing University of Duhok Duhok Kurdistan Region Iraq; ^3^ College of Medicine University of Duhok Duhok Kurdistan Region Iraq; ^4^ Department of Health and Caring Sciences University of Gothenburg Gothenburg Sweden

## Abstract

**Background and Aims:**

Venipuncture and cannulation are common yet distressing procedures for children in healthcare settings. While both are known to cause significant pain and fear, prior research has examined them in isolation, leaving a critical gap: no study has directly compared the distress profiles of these two procedures. This study aimed to directly assess and compare the levels of pain and fear reported by pediatric patients undergoing venipuncture versus cannulation.

**Methods:**

This prospective cross‐sectional study compared pain and fear levels among 298 pediatric patients aged 6–12 years who underwent venipuncture or cannulation at a pediatric teaching hospital. Pain was assessed using the Wong–Baker FACES pain rating scale, and fear was measured with the Children's fear scale (CFS); the number of procedure attempts and duration were also recorded.

**Results:**

Patients in the cannulation group experienced significantly higher mean pain scores during (7.09 vs. 6.55, *p* = 0.012) and after (7.12 vs. 6.65, *p* = 0.029) the procedure than those in the venipuncture group. Fear levels were also elevated in the cannulation group during (3.16 vs. 2.82, *p* < 0.001) and after (3.12 vs. 2.77, *p* < 0.001) the procedures. Cannulation was associated with more procedure attempts (1.61 vs. 1.42, *p* < 0.001) and longer durations (5.06 vs. 4.11 min, *p* < 0.001) than venipuncture.

**Conclusions:**

This study found that pediatric patients experience significantly greater pain and fear during cannulation procedures than during venipuncture procedures, which may be related to variations in invasiveness, anatomical location, procedure duration, and number of attempts required.

## 1 Introduction

Venipuncture and cannulation are among the most frequently performed invasive procedures in pediatric healthcare settings. Venipuncture involves inserting a needle into a vein for blood sampling, while cannulation involves placing an intravenous catheter for the administration of fluids, medications, or blood products. Both procedures are indispensable to the diagnosis and treatment of children across a wide range of clinical conditions. Although considered routine components of pediatric care, their execution requires trained healthcare professionals—including nurses and physicians—who must balance precise technical performance with careful attention to the child's comfort and psychological well‐being [1].

Despite their clinical indispensability, these procedures represent a significant source of fear, pain, and apprehension for many pediatric patients [2]. The psychological burden associated with these procedures is not confined to the immediate clinical encounter. In the short term, procedural distress manifests as acute fear and pain that can compromise patient cooperation and procedural success [3, 4, 5]. Over time, repeated or poorly managed procedural experiences may contribute to the development of persistent fear of clinical environments, healthcare avoidance behaviors, and heightened pain sensitivity—consequences with meaningful implications for long‐term health engagement [3, 4, 5].

Research consistently demonstrates that venipuncture is associated with moderate to severe pain in a substantial proportion of pediatric patients [6, 7]. Similarly, the insertion of an intravenous cannula is recognized as a significant source of procedural discomfort and distress [8]. Despite the shared needle‐based nature of these two procedures, they differ meaningfully in their technical complexity, the degree of tissue disruption involved, the placement of an indwelling device in the case of cannulation, and the number of attempts typically required. The existing literature has largely explored these procedures independently or evaluated the effectiveness of interventions—including non‐pharmacological approaches such as virtual reality—in reducing procedural distress associated with needle procedures more broadly [9, 10], rather than directly comparing the distress profiles of venipuncture and cannulation. These procedural differences provide an a priori basis for expecting that the intensity and character of pain and fear may differ between them—and, by extension, that the management strategies required may also need to be procedure‐specific. For instance, the greater invasiveness and longer duration of cannulation may necessitate more intensive or multimodal approaches to distress management, including topical anesthesia and structured non‐pharmacological interventions, compared with venipuncture.

Effective procedural pain management is fundamental to the physical and psychological well‐being of pediatric patients [7, 11]. Unmanaged pain and fear can precipitate procedural failure, increased healthcare costs, and prolonged hospital stays [3]. Despite this, a critical gap remains in the literature: to date, no study has directly and quantitatively compared pain and fear experiences of pediatric patients undergoing venipuncture versus cannulation. Understanding whether, and to what degree, these procedures differ in the distress they produce is essential for developing evidence‐based, procedure‐specific approaches to pain and fear management in this population [4].

Previous comparative research has been limited by a focus either on a single procedure type or on specific interventions, without characterizing baseline differences in procedural distress between venipuncture and cannulation. A direct, empirical comparison is therefore needed to inform tailored clinical protocols that are commensurate with the specific procedural burden imposed on the child.

This study aimed to directly assess and compare the severity of pain and the experience of fear in pediatric patients undergoing venipuncture versus cannulation procedures, to optimize procedure‐specific pain and fear management strategies and improve the overall healthcare experience for pediatric patients.

## 2 Method

### 2.1 Study Design and Setting

For this study, a prospective cross‐sectional design was selected. The reporting of this study adheres to the Strengthening the Reporting of Observational Studies in Epidemiology (STROBE) guidelines for cross‐sectional studies [12]. The research was conducted at Heevi Pediatric Teaching Hospital, the main pediatric healthcare facility in the Duhok Province of Iraqi Kurdistan. This hospital provides comprehensive healthcare services to children across various units, including the Emergency Department (ED), Pediatric Intensive Care Unit (PICU), medical wards, operating room, surgical ward, and intensive care unit. The patients were recruited over 3 months, from December 2023 to March 2024.

### 2.2 Participants

This study involved pediatric patients and the nurses who performed the procedures. Patient recruitment was conducted via consecutive sampling from the emergency department of Heevi Pediatric Teaching Hospital. All children aged 6 to 12 years presenting to the emergency department during the study period who met the inclusion criteria and were scheduled for either venipuncture or cannulation procedures were approached for participation in the order of their presentation until the target sample size was reached. Informed consent was obtained from parents or legal guardians, and when appropriate, assent was also obtained from the children. To ensure a focused study population, the following exclusion criteria were applied: children with chronic/complex diseases (e.g., sickle cell disease, renal failure) or a history of chemotherapy; children with children with cognitive or communicative impairments; children who had taken analgesics within the preceding 6 h; individuals outside the 6–12‐year age range; and those who had undergone a similar procedure during the current hospitalization.

Two registered pediatric nurses performed the procedures. To minimize operator bias, both nurses were selected based on standardized high‐level expertise, each possessing over 5 years of clinical experience in the hospital's emergency department and formal credentialing to perform both venipuncture and cannulation in children. They received identical brief retraining on the study protocol prior to data collection, emphasizing adherence to the hospital's standard operating procedures for each technique (including site preparation, needle gauge selection, and child engagement). Patient assignment was based solely on the clinical indication; the nurse specialized in venipuncture performed all venipunctures, and the nurse specialized in cannulation performed all cannulations, mirroring the real‐world clinical workflow.

### 2.3 Procedure Protocol

All venipuncture and cannulation procedures were performed in accordance with the hospital's standardized pediatric venous access protocol. Prior to the procedure, the child was positioned comfortably, and the site was selected based on clinical appropriateness: venipuncture primarily targeted the antecubital fossa veins, whereas cannulation preferentially utilized the superficial dorsal hand veins. Following the emergency department's standard operating procedures at the time of the study—where rapid vascular access is often prioritized—topical local anesthetics were not routinely administered. The skin was prepared using a 70% isopropyl alcohol swab with a circular motion and allowed to dry. Venipuncture was performed using a sterile safety blood collection needle (typically 21–23 gauge), and cannulation was performed using a sterile intravenous safety catheter (typically 22–24 gauge). Both procedures were conducted using a standardized technique: vein stabilization, bevel‐up insertion at a 15°–30° angle, and confirmation of blood flashback before sample collection or catheter advancement. Following successful access, venipuncture sites received manual pressure with sterile gauze, while cannulas were secured with a transparent sterile dressing and flushed with 0.9% saline to ensure patency unless clinically contraindicated. All other aspects of patient preparation, positioning, and comfort (e.g., use of a parent for comfort holding, age‐appropriate explanation) were performed in accordance with nurses' standard clinical practice.

### 2.4 Sample Size

A formal a priori power calculation was not feasible due to the absence of prior data on the expected magnitude of difference in pain scores between pediatric venipuncture and cannulation—the primary novel comparison of this study. Therefore, the initial sample size was estimated pragmatically using Slovin's formula for finite populations (*n* = *N*/(1 + Ne^2^)), a method commonly used in pediatric procedural studies in our regional context to determine robust sample sizes for group comparisons [13, 14]. Based on the annual patient volume for the target age group (6–12 years) at the study hospital and a margin of error (*e*) of 0.05, the calculation yielded a target of approximately 286. This was increased to 300 to account for potential incomplete data or withdrawal.

To objectively confirm the adequacy of the final enrolled sample (*N* = 298) for the planned comparative analyses, a post‐hoc sensitivity analysis was conducted using G*Power 3.1. With the obtained group sizes (Venipuncture, *n* = 150; Cannulation, *n* = 148), an alpha of 0.05, and power (1 − β) of 0.80, the analysis indicated that the study was sensitive enough to detect a small‐to‐medium effect size (Cohen's *d* = 0.326) for between‐group differences. The observed effect sizes for the primary outcomes approached or exceeded this threshold, confirming that the sample provided sufficient statistical power for the key comparisons.

### 2.5 Data Collection and Procedure

Data collection was performed by a dedicated research assistant to ensure consistency. To reflect standard clinical practice and minimize measurement bias, a strict role separation was implemented. One specialized pediatric nurse performed all venipuncture procedures, while a second, different nurse performed all cannulation procedures. This design reflects pragmatic clinical practice, where nurses often specialize in specific procedures.

The research assistant, who was blinded to the study's specific comparative hypotheses, collected all outcome data using a standardized protocol. Immediately after each procedure, the child's physiological parameters—respiration rate, heart rate, and peripheral oxygen saturation—were recorded using calibrated bedside monitors. The child then self‐reported pain and fear using the Wong–Baker FACES pain rating scale and the Children's fear scale, presented with identical, scripted instructions. This design ensured that the data collector did not influence the clinical procedure, reducing the potential for assessment bias.

### 2.6 Measures

#### 2.6.1 Demographic and Clinical Characteristics Form

This form included age, sex, previous pain, previous fear, procedure history, needle gauge, and use of pharmacological and non‐pharmacological interventions.

#### 2.6.2 Children's Pain Experience

In our study, we utilized the Wong–Baker FACES pain rating scale to assess children's pain experiences. This scale presents different faces, ranging from smiling to crying, and children choose the face that best represents the intensity of their pain, assigning it a score from 0 to 10. Following the medical procedure, we instructed the children to indicate the face that matched their perceived pain level. This standardized approach enabled us to systematically measure and comprehend patients' pain experiences, ensuring the reliability and scientific integrity of our study [15].

#### 2.6.3 Children's Fear Experience

We assessed fear using the Children's Fear Scale (CFS), a validated and reliable tool designed for evaluating fear in young children aged 5–10 years. The scale consists of five faces evenly distributed along a horizontal axis, each depicting a different fear expression. The faces are assigned numerical values, with the one on the far right marked as number 4, indicating the highest level of fear, and the face on the far left marked as number 0, representing no fear at all. The children were provided with a pen to indicate their most appropriate face on the scale [16]. As a single‐item measure, the CFS is not amenable to assessment of internal consistency using Cronbach's alpha. Instead, reliability was evaluated by calculating the intraclass correlation coefficient (ICC; two‐way mixed, absolute agreement, single measures) between CFS scores obtained during and after the procedure. The ICC was 0.721 (95% CI: 0.649–0.778), indicating good consistency in fear ratings across the two assessment periods and supporting the scale's reliability in the current sample.

### 2.7 Statistical Analysis

Data management and analysis were performed using IBM SPSS Statistics for Windows (Version 26.0). Descriptive statistics are presented as mean and standard deviation [17] for continuous variables, and as frequency and percentage for categorical variables. All inferential analyses were prespecified in the study protocol to test the primary hypothesis comparing venipuncture and cannulation groups. No exploratory or subgroup analyses were conducted.

The normality of distribution for continuous outcome variables was assessed within each procedure group using visual inspection of Q–Q plots and histograms. Homogeneity of variances was confirmed using Levene's test. Between‐group comparisons (venipuncture vs. cannulation) for normally distributed continuous outcomes—including pain scores, fear scores, physiological parameters, number of attempts, and procedure duration—were conducted using independent samples *t*‐tests. Group comparisons for the categorical variable (sex) were performed using the Pearson Chi‐squared test.

For all comparative tests, effect sizes were calculated and reported as Cohen's d. The magnitude of Cohen's d was interpreted as small (0.2), medium (0.5), and large (0.8) based on standard conventions [18]. Ninety‐five percent confidence intervals (95% CI) are reported for mean differences and key effect sizes. The threshold for statistical significance was set a priori at a two‐sided alpha (α) level of 0.05; however, *p*‐values are interpreted in the context of effect sizes and confidence intervals to avoid over‐reliance on dichotomous significance testing.

### 2.8 Ethical Considerations

This study was conducted in accordance with the ethical principles for medical research involving human subjects outlined in the Declaration of Helsinki [19]. The study protocol received formal approval from the Health Ethics Committee of the Directorate General of Health‐Duhok (reference number: 12072017‐5) in December 2023 prior to commencement.

Written informed consent was obtained from a parent or legal guardian for all participating children. Age‐appropriate written assent was also obtained from the children themselves whenever possible. For the two nurses performing the procedures, written informed consent was obtained after a full explanation of their role. A fundamental principle was respect for autonomy; the nurses were explicitly informed of their right to decline or withdraw participation at any time. To protect their privacy, no personally identifiable information of the nurses was recorded on any study materials.

## 3 Results

### 3.1 Patient Characteristics

A total of 298 pediatric patients participated in the study, with 150 undergoing venipuncture and 148 undergoing cannulation. The demographic and baseline characteristics of the two groups were comparable (Table 1). The mean age was similar between the venipuncture group (8.94 years, SD = 1.43) and the cannulation group (8.96 years, SD = 1.47), with a mean difference of −0.02 years (95% CI: −0.35 to 0.31, *p* = 0.908). Sex distribution was similar between groups, with 66 of 150 patients (44.0%) in the venipuncture group and 69 of 148 patients (46.6%) in the cannulation group identified as male (*p* = 0.727). Although the cannulation group reported slightly higher mean pain and fear scores from previous procedures, these differences were not statistically significant (Pain: mean difference = 0.21, 95% CI: −0.06 to 0.46, *p* = 0.128; Fear: mean difference = 0.16, 95% CI: −0.01 to 0.34, *p* = 0.070).

**Table 1:** Comparison of general features between the venipuncture and cannulation groups.

Patients' characteristics	Venipuncture group (*n* = 150)	Cannulation group (*n* = 148)	*p*‐value (Two‐sided)	Mean diff (95% CI)
Age (year), mean (SD)	8.94 (1.425)	8.96 (1.466)	0.908^a^	−0.349 to 0.310
Gender, *n* (%)				
Male	66 (48.9%)	69 (51.1%)	0.727^b^	
Female	84 (51.5%)	79 (48.5%)		
Pain previous procedure, mean (SD)	6.93 (1.213)	7.135 (1.060)	0.128^a^	−0.461 to 0.058
Fear previous procedure, mean (SD)	2.95 (0.809)	3.11 (0.720)	0.070^a^	−0.336 to 0.013

Abbreviations: CI, confidence interval; M, Mean; SD, standard deviation.

^a^ An independent *t*‐test.

^b^ Pearson Chi‐squared test was performed for statistical analyses.

### 3.2 Comparison of Pain and Fear Scores

Children undergoing cannulation reported significantly higher levels of pain and fear compared to those undergoing venipuncture (Table 2). During the procedure, mean pain scores were higher in the cannulation group (7.09, SD = 1.90) than in the venipuncture group (6.55, SD = 1.77), with a mean difference of 0.54 (95% CI: 0.12 to 0.96, *p* = 0.012, Cohen's *d* = 0.26). This difference persisted after the procedure (Cannulation: 7.12, SD = 1.92; Venipuncture: 6.65, SD = 1.75; mean difference = 0.47, 95% CI: 0.05 to 0.89, *p* = 0.029, Cohen's *d* = 0.30).

**Table 2:** Comparison of pain and anxiety scores between venipuncture and cannulation groups.

Patients' outcomes	Venipuncture group (*n* = 150), M (SD)	Cannulation group (*n* = 148)	*p*‐value (Two‐sided)	Mean diff (95% CI)	Effect size (Cohen's *d*)
Pain during procedure	6.55 (1.771)	7.09 (1.902)	0.012	−0.960 to 0.122	0.26
Pain post procedure	6.65 (1.749)	7.12 (1.923)	0.029	−0.887 to 0.049	0.30
Fear during procedure	2.82 (0.751)	3.16 (0.700)	< 0.001	−0.508 to 0.177	0.46
Fear post procedures	2.77 (0.798)	3.12 (0.699)	< 0.001	−0.526 to 0.184	0.47

*Note:* An independent *t*‐test was performed for statistical analyses. Pain intensity was measured by Wong–Baker FACES Pain Rating Scale (WBFS). Fear levels were measured by the Children's Fear Scale (CFS).

Abbreviations: CI, confidence interval; M, Mean; SD, standard deviation.

Fear levels followed a similar pattern. During the procedure, the cannulation group reported a mean fear score of 3.16 (SD = 0.70) compared with 2.82 (SD = 0.75) in the venipuncture group, a mean difference of 0.34 (95% CI: 0.18 to 0.51, *p* < 0.001, Cohen's *d* = 0.46). Post‐procedure fear scores were also significantly higher for cannulation (3.12, SD = 0.70 vs. 2.77, SD = 0.80; mean difference = 0.35, 95% CI: 0.18 to 0.53, *p* < 0.001, Cohen's *d* = 0.47).

### 3.3 Procedural Characteristics: Attempts and Duration

Cannulation procedures required more attempts and longer durations than venipuncture (Table 3). The mean number of attempts required for successful cannulation was 1.61 (SD = 0.49), significantly higher than the 1.42 attempts (SD = 0.50) for venipuncture (mean difference = 0.19, 95% CI: 0.08 to 0.31, *p* < 0.001, Cohen's *d* = 0.40). The mean duration of the cannulation procedure was 5.06 min (SD = 0.68), which was nearly 1 min longer than the mean duration of venipuncture (4.11 min, SD = 0.67; mean difference = 0.95, 95% CI: 0.80 to 1.11, *p* < 0.001, Cohen's *d* = 1.40).

**Table 3:** Comparison of number of attempts and duration between venipuncture and cannulation groups.

Procedures	Venipuncture group (*n* = 150) M (SD)	Cannulation group (*n* = 148) M (SD)	*p*‐value (Two‐sided)	Mean diff (95% CI)	Effect size (Cohen's *d*)
Number of attempts	1.42 (0.495)	1.61 (0.488)	< 0.001	−0.307 to 0.083	0.40
Duration (min)	4.11 (0.670)	5.06 (0.676)	< 0.001	−1.107 to 0.800	1.40

*Note:* An independent *t*‐test was performed for statistical analyses.

Abbreviations: CI, confidence interval; M, Mean; SD, standard deviation.

### 3.4 Post‐Procedural Physiological Parameters

Physiological parameters measured immediately after the procedure also differed between the two groups (Table 4). Children in the cannulation group exhibited a significantly higher mean respiration rate (21.14 breaths/min, SD = 0.92) compared to the venipuncture group (20.07 breaths/min, SD = 1.05), with a mean difference of 1.07 breaths/min (95% CI: 0.84 to 1.29, *p* < 0.001, Cohen's *d* = 1.08). Heart rate was also higher in the cannulation group (92.91 beats/min, SD = 1.01) than in the venipuncture group (91.04 beats/min, SD = 0.97), a mean difference of 1.87 beats/min (95% CI: 1.64 to 2.09, *p* < 0.001, Cohen's *d* = 1.88). Oxygen saturation (SpO_2_) was slightly but significantly higher in the cannulation group (98.16%, SD = 1.06) compared to the venipuncture group (97.89%, SD = 1.11), with a mean difference of 0.28% (95% CI: 0.03 to 0.52, *p* = 0.029, Cohen's *d* = 0.25).

**Table 4:** Comparison of physiological parameters measured immediately after the procedure.

Physiological parameter	Venipuncture group (*n* = 150)	Cannulation group (*n* = 148)	Mean difference (95% CI)	*p*‐value	Effect size (Cohen's *d*)
Respiration rate (breaths/min)	20.07 (1.05)	21.14 (0.92)	−1.07 (−1.29 to −0.84)	< 0.001	−1.08
Heart rate (beats/min)	91.04 (0.97)	92.91 (1.01)	−1.87 (−2.09 to −1.64)	< 0.001	−1.88
Oxygen saturation, SpO_2_ (%)	97.89 (1.11)	98.16 (1.06)	−0.28 (−0.52 to −0.03)	0.029	−0.25

*Note:* Data are presented as CI, confidence interval; M, mean; SD, standard deviation. An independent *t*‐test was performed for statistical analyses.

## 4 Discussion

This study provides the first direct comparative evidence quantifying a significant disparity in procedural distress between two fundamental nursing interventions: peripheral intravenous cannulation induces markedly greater pain and fear in pediatric patients than venipuncture. The elevation of distress metrics both during and following cannulation supports its characterization as a more aversive clinical event and suggests that it may represent a critical priority for targeted nursing intervention [5, 20, 21, 22].

### 4.1 Interpreting the Findings

#### 4.1.1 Consideration of Contextual and Confounding Variables

The present analysis compared these procedures as integrated clinical entities, where technique, customary anatomical site, and device gauge constitute an inseparable experiential bundle. While venipuncture typically employs a finer‐gauge needle on superficial dorsal hand veins, cannulation commonly involves a larger catheter in deeper antecubital vessels [23, 24, 25, 26, 27]. These technically embedded variables, though recorded, were not isolated in this pragmatic design. This approach strengthens ecological validity, as nursing care addresses the holistic procedure. Future research may employ factorial designs to disentangle the specific contribution of site and gauge.

#### 4.1.2 Interpretation of Observed Associations

The heightened distress observed in the cannulation group may reflect a convergence of physical and psychological factors that differ between the two procedures. Anatomically, the cannulation procedure often involves different locations than venipuncture, which could potentially influence nociceptive input due to variations in tissue density or nerve distribution [28, 29]. Procedurally, our data indicate that cannulation was associated with a longer mean duration (by nearly 1 min) and a greater number of attempts than venipuncture. This prolonged exposure and increased technical complexity may plausibly contribute to higher anticipatory fear, particularly among older children who may better comprehend the implications of an indwelling device [16, 22].

While the observed associations between procedural difficulty, duration, characteristics, and distress are broadly consistent with contemporary biopsychosocial models of pediatric pain and fear [30, 31], further research is needed to understand better the potential pathways underlying these differences.

#### 4.1.3 Subjective Experience Versus Physiological Arousal

A critical finding is the marked discordance in effect magnitudes between subjective and objective distress metrics. Participants undergoing cannulation exhibited profound autonomic arousal, as evidenced by a marked increases in heart rate and respiratory rate. In contrast, the increments in self‐reported pain and fear, while statistically significant, were associated with small‐to‐moderate effect sizes. This divergence may reflect the constraints of self‐report tools, developmental differences in affective expression, or the distinction between conscious perception and neurovegetative activation [32]. For nursing assessment, this highlights an important principle: objective physiological parameters, particularly cardiorespiratory indices, may serve as essential and complementary biomarkers of acute distress, supporting their integration with—and occasional primacy over—subjective report.

### 4.2 Implications for Nursing Theory and Practice

These empirical findings resonate with core nursing theory. The significant deficit in patient comfort during cannulation aligns with Kolcaba's Theory of Comfort, suggesting substantive gaps in the physical (pain) and psychospiritual (fear) dimensions [33, 34]. Consequently, these results suggest a clinical imperative: the documented disparity suggests the value of differentiated procedural care standards.

This supports the development of a structured, enhanced comfort protocol for pediatric cannulation that is distinct from venipuncture care. Such a protocol should be multimodal, proactively integrating evidence‐based pharmacological and non‐pharmacological strategies [35]. Furthermore, institutional policy should prioritize first‐attempt success by aligning the highest clinician expertise with this more distressing procedure [36, 37, 38]. This evidence offers a foundation for nursing leadership to advocate for a tiered approach to procedural support, ensuring the nursing response is commensurate with the procedural burden.

### 4.3 Study Strengths and Limitations

#### 4.3.1 Strengths

Methodological strengths include the prospective comparative design, use of validated child‐centered instruments, and a substantial sample size (*N* = 298), which bolsters internal validity. A post‐hoc sensitivity analysis confirmed adequate power to detect the observed effects. The implementation of blinded outcome assessment minimizes detection bias, while the real‐world setting with specialized nurse operators ensures high ecological validity and direct translational relevance.

#### 4.3.2 Limitations

Interpretation is tempered by several limitations. First, the single‐center, cross‐sectional design may constrain the generalizability of findings to other healthcare settings or cultural contexts. Second, while consecutive sampling was used, residual confounding from unmeasured variables (e.g., child temperament) cannot be excluded. Third, reliance on self‐reported measures, though standard, carries inherent limitations, and the presence of research personnel may have introduced observer bias (the Hawthorne effect). Finally, and most critically in the context of mechanistic interpretation, the observational design limits causal inference regarding the procedure type itself. Future multisite studies are needed to confirm these findings and explore moderating factors such as developmental stage.

## 5 Conclusion

This comparative study found that pediatric peripheral intravenous cannulation elicits significantly greater pain and fear than venipuncture, which may be related to procedural characteristics including invasive nature, longer duration, and greater number of attempts. These findings highlight the necessity for enhanced, procedure‐specific pain and fear management protocols in clinical practice to improve the procedural experience for children.

## Author Contributions

**Sherzad Khudeida Suleman:** methodology, validation, formal analysis, data curation, writing – original draft, writing – review and editing, investigation, conceptualization, project administration. **Nizar BakirYahya:** conceptualization, methodology, formal analysis;, investigation, supervision, resources, writing – review and editing. **Stefan Nilsson:** conceptualization, methodology, investigation, writing – review and editing, formal analysis, supervision. **Karin Enskär:** conceptualization, methodology, validation, supervision, formal analysis, writing – original draft, writing – review and editing, investigation.

## Funding

The authors have nothing to report.

## Disclosure

Corresponding author “Sherzad Khudeida Suleman” affirms that this manuscript is an honest, accurate, and transparent account of the study being reported; that no important aspects of the study have been omitted; and that any discrepancies from the study as planned have been explained.

## Conflicts of Interest

The authors declare no conflicts of interest.

## Data Availability

The data that support the findings of this study are available on request from the corresponding author. The data are not publicly available due to privacy or ethical restrictions.
